# Magnetic Recording Method (MRM) for Nondestructive Evaluation of Ferromagnetic Materials

**DOI:** 10.3390/ma15020630

**Published:** 2022-01-14

**Authors:** Tomasz Chady, Ryszard D. Łukaszuk, Krzysztof Gorący, Marek J. Żwir

**Affiliations:** 1 Faculty of Electrical Engineering, West Pomeranian University of Technology, 70-313 Szczecin, Poland; 2 Doctoral School, West Pomeranian University of Technology, 70-313 Szczecin, Poland; ryszard.lukaszuk@zut.edu.pl; 3 Department of Polymer and Biomaterials Science, Faculty of Chemical Technology and Engineering, West Pomeranian University of Technology, 70-311 Szczecin, Poland; krzysztof.goracy@zut.edu.pl (K.G.); marek.zwir@zut.edu.pl (M.J.Ż.)

**Keywords:** nondestructive testing (NDT), nondestructive evaluation (NDE), magnetic recording method (MRM), ferromagnetic materials, stress test, structural health monitoring (SHM)

## Abstract

This paper proposes and experimentally investigates a novel nondestructive testing method for ferromagnetic elements monitoring, the Magnetic Recording Method (MRM). In this method, the inspected element must be magnetized in a strictly defined manner before operation. This can be achieved using an array of permanent magnets arranged to produce a quasi-sinusoidal magnetization path. The magnetic field caused by the original residual magnetization of the element is measured and stored for future reference. After the operation or loading, the magnetic field measurement is repeated. Analysis of relative changes in the magnetic field (for selected components) allows identifying applied stress. The proposed research methodology aims to provide information on the steel structure condition unambiguously and accurately. An interpretation of the results without referring to the original magnetization is also possible but could be less accurate. The method can be used as a standard technique for NDT (Non-Destructive Testing) or in structural health monitoring (SHM) systems.

## 1. Introduction

The need to reduce greenhouse gas emissions and, due to the Paris Agreement, the need for countries to achieve climate neutrality in the second half of the 21st century have resulted in modifications to structural components. One such change is the production of components with a reduced thickness or cross-sectional area. However, the negative effect of such an approach is the significant impact of even small heterogeneities on the structural strength of the part, which may threaten the safe use of the structure. Therefore, it is necessary to frequently evaluate the structure with nondestructive testing.

Carbon structural steels are the primary construction materials that have a specific chemical composition defined for these varieties, and are delivered in the form of sheets and other rolled products with fixed, typical cross-sections. The chemical composition of structural carbon steels is designed for their intended use. In Europe, the requirements for such steels are specified in the European standard EN 10025. Examples of carbon structural steels are S195, S235, S355, S420, and S460. The letter S in the steel designation indicates “carbon structural steel” and the number following it specifies the minimum yield stress for this steel grade in MPa. The EN 10025 standard defines the yield stress as a value at which irreversible plastic deformation of a rod with a diameter of 16 mm will occur.

In engineering practice, the yield strength is a point on the graph of stress dependence on the strain, which means exceeding the stresses below, with material behaving according to Hook’s law. That is, if the stress does not exceed the yield strength, the material behaves perfectly elastic. After exceeding the yield strength, at least part of the deformation of the material will be permanent. The yield strength is a number characteristic for a given material. In practice, it means the maximum stress that a part or structure can carry without permanent damage. For structural carbon steels, this limit is relatively easy to determine.

Carbon structural steels are ferromagnetic and retain their ferromagnetic properties up to a temperature of about 770 °C—in this respect, they have properties such as their main component, iron. This distinguishes them from alloy steels in which the Curie temperature strongly depends on other alloying elements present in their composition: Ni, Cr, Mn, Co. This dependency in some configurations of constituents may even lead to the loss of ferromagnetic properties at ambient temperature (e.g., austenitic steels).

The conditions of magnetic materials can be examined in a nondestructive way using the following methods:

The magnetic flux leakage method relies on analyzing changes in the magnetic field distribution around the tested object. Magnetizing the material with an external magnetic field excites the magnetic flux in the material. If the flux encounters any geometrical inhomogeneities with significantly lower permeance, it breaks out of the material and can be registered by the magnetic sensor [1,2]. Flux leakage allows the inspector to localize and identify surface and subsurface flaws [3]. The inevitable advantages of this technique are high efficiency and no requirement for direct contact with the tested object [4,5]. However, it also has some disadvantages, such as susceptibility to the flaw orientation, the need to demagnetize the object after inspection, a sensitivity that is dependent on the distance between sensor and material, and difficulty detecting small and stress-induced changes [6,7,8].

The Barkhausen noise method is based on the phenomenon occurring in ferromagnetic material. The structure of any such material is made up of magnetic domains separated by domain walls. Each domain contains dipoles oriented in one privileged magnetization direction [9,10]. The external magnetic field will cause the movement of the domain walls. If any inhomogeneities occur in the material’s internal structure, the walls change their position discontinuously. This process is accompanied by a sudden change in magnetization and an induction of voltage pulses in the sensor coil [11]. This technique is suitable for detecting surface and subsurface changes, determining grain dimensions or hardness, and assessing stress levels [12,13]. Some benefits of this method include good sensitivity, a simple examination procedure, no requirement for surface preparation, and quick residual stress recognition [14,15]. This method suffers several drawbacks: the necessity of sensor calibration and a non-standardized measurement approach [16,17].

The Magnetic Memory Method is a relatively novel approach to the nondestructive inspection of ferromagnetic materials. It was proposed by Dubov in 1997 [18]. Under the influence of Earth’s magnetic field or applied stress, the intrinsic magnetic domains irreversibly change their position and direction [19]. The process of stress influence on magnetic materials has been known for a long time as an inverse magnetostrictive effect or Villari effect [20]. At the core of the metal magnetic memory method is the detection of a self-magnetic leakage field, indicating the inhomogeneities of the internal structure caused by the effect mentioned above [21]. The significant advantages of this method are no requirement to prepare the surface or premagnetize or demagnetize the material, low-cost measurement equipment, simplicity, time-saving inspection procedure, and the possibility to detect and localize the stress zones, thus avoiding a sudden catastrophic accident [22,23,24,25]. The disadvantages of this technique include a weak field forcing the use of sensitive sensors and its applicability only if no external, strong magnetic fields act on the material before or during the inspection [25,26].

## 2. Materials and Methods

The proposed new method for nondestructive testing of magnetic materials is somehow like those discussed in Section 1, particularly the magnetic memory method.

In the case of the proposed Magnetic Recording Method (MRM), the tested object has to be magnetized in a strictly defined way, e.g., quasi-triangular or quasi-sinusoidal pattern. If external factors such as static stresses act on the material, the residual magnetization changes. By analyzing changes in the magnetic field caused by residual magnetization, it is possible to determine the intensity and direction of the structural influences.

The samples used in the experiments were made of structural S355 steel. Due to its beneficial properties and low-cost production, S355 is widely used in modern industry branches such as civil engineering, offshore, shipbuilding, and automotive [27,28,29,30,31,32,33,34]. The chemical composition of S355 is as follows: Mn—1.45, Al—0.33, P—0.23, Si—0.21, C—0.17, S—0.08 [32]. The exemplary magnetic properties of the steel S355 are as follows [35]: a relative peak permeability of 1500, a saturation point of 1.7 T at 6.9 kA/m, a coercive field of 310 A/m, and a residual flux density of 1 T (measured on the major loop).

**Figure 1:**
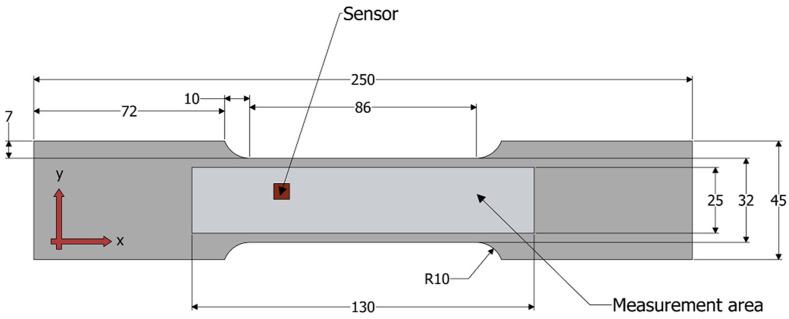
Sample shape and dimensions with depicted measurement area.

Each sample was cut out of a hot-rolled plate using a waterjet cutter to avoid jagged metal edges. The shape and dimensions of the samples produced in this way are shown in Figure 1.

**Figure 2:**
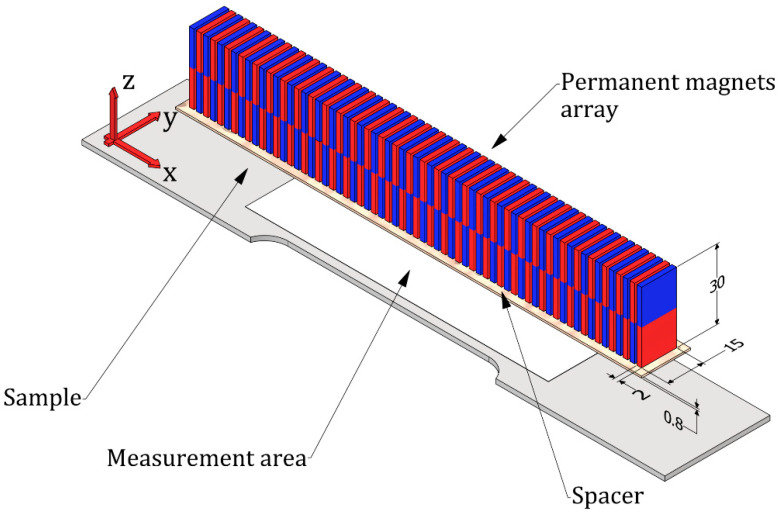
The array of magnets over the sample under magnetization.

The measuring procedure consisted of four steps. In the first step, the sample was magnetized in a strictly defined manner. The magnetizing element consisted of the magnets configured in the array to generate a quasi-sinusoidal magnetization pattern in the sample. A simplified view of the magnetizing element is shown in Figure 2. It was constructed using 100 neodymium plate magnets, 2 mm high, 15 mm wide, and 30 mm long, made of N38 material, and magnetized in the length direction (30 mm). The material N38 (Nd_2_Fe_14_B) has the following magnetic parameters: remanence *B_r_* = 1.2 T; coercivity *H_cb_* ≥ 899 kA/m; coercivity *H_cJ_* ≥ 955 kA/m; energy density (*BH*) max ≥ 287–310 kJ/m^3^. The magnets were separated from each other with a tape 0.12 mm thick. On one side (facing the magnetized sample), a 0.8 mm thick PTFE (polytetrafluoroethylene) spacer was glued to the array of magnets to facilitate sliding and ensure a permanent lift-off. The magnetic field in the gap between the magnets and the magnetized sample was 0.97 T. It was measured with a GM08 Gaussmeter manufactured by Hirst Magnetic Instruments (Falmouth, United Kingdom) with a PT7810 Hall effect probe. The array was manually moved above the sample surface with a lift-off of 0.8 mm in a direction parallel to the *y*-axis from one edge to the other edge of the sample (Figure 2). The magnets were moved at a speed of around 5 mm/s. In this way, the plate was magnetized relatively evenly in the *y*-axis direction. If necessary, the uniformity of the magnetization could be improved by using a motorized mechanical scanner.

**Figure 3:**
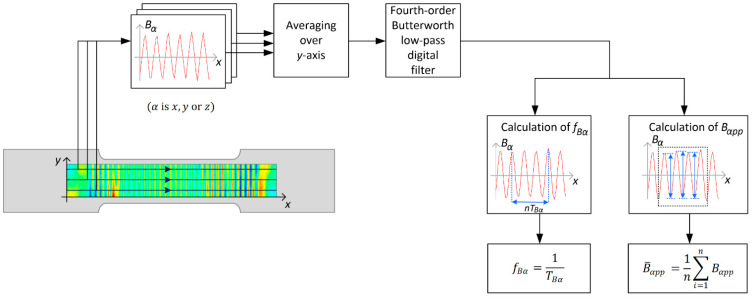
The measuring procedure.

In the second step, the magnetic field caused by the residual magnetization of the sample was measured with a three-axis magnetometer (HMC5883L) moved in the *x*- and *y*-directions over the sample surface (lift-off 0.3 mm) in the area depicted in Figure 1 and Figure 2. The third step of the procedure included filtering two-dimensional signals and then averaging, which results in obtaining one-dimensional signals. In the last stage, one-dimensional signals were analyzed and their characteristic parameters, such as amplitude and frequency, were determined. A flowchart of the procedure designated for this purpose is shown in Figure 3.

In all cases, data measured for selected *y*-coordinates were used for the analysis. The scanning paths were chosen in such a way as to avoid the influence of the edge effect on the calculation of characteristic parameters. The selected signals were used to calculate an average signal. Next, a low-pass, fourth-order, digital Butterworth filter ($f/f_{N}=0.4$, $f_{N}$—Nyquist frequency) was used to remove external interferences of the measured signals. After filtration, the characteristic parameters of the signal were calculated. Several cycles of the signal were selected to determine the signal period, and thus its frequency (Equation (1)):

(1) $$f_{B\alpha }=\frac{1}{T_{B\alpha }}$$

where: α—*x*, *y,* or *z* component of the magnetic field, $f_{B\alpha }$—frequency of a given magnetic field component, and $T_{B\alpha }$—magnetic field period of a given component. Then, the windowed central part of the signal (corresponding to the magnetic field measured in the middle part of the sample) was utilized to calculate the mean peak-to-peak value (Equation (2)):

(2) $${\bar{B}}_{\alpha pp}=\frac{1}{n}\overset{n}{\underset{i=1}{\sum }}B_{\alpha pp}$$

where: $B_{\alpha pp}$—the peak-to-peak value of magnetic field component (α could be *x*, *y,* or *z*), *n*—the number utilized in calculations of peak-to-peak values of $B_{\alpha }$, ${\bar{B}}_{\alpha pp}$—mean peak-to-peak value of magnetic field component.

Furthermore, additional calculations: relative mean change in magnetic field (Equation (3)) and relative frequency change in the magnetic field (Equation (4)) were performed to assess the variations in magnetization of the samples after their stress-loading.

(3) $$\Delta {\bar{B}}_{\alpha }=\frac{{\bar{B}}_{\alpha pp}^{before}-{\bar{B}}_{\alpha pp}^{after}}{{\bar{B}}_{\alpha pp}^{before}}\cdot 100\%$$

(4) $$\Delta f_{B\alpha }=\frac{f_{B\alpha }^{before}-f_{B\alpha }^{after}}{f_{B\alpha }^{before}}\cdot 100\%$$

where: α—could be *x*, *y,* or *z* component of the magnetic field, ${\bar{B}}_{\alpha pp}^{before}$—mean peak-to-peak value of the magnetic field for the non-stressed samples, ${\bar{B}}_{\alpha pp}^{after}$—mean peak-to-peak value of the magnetic field for the samples after tensile loading, $f_{B\alpha }^{before}$—signal frequency for the non-stressed samples, $f_{B\alpha }^{after}$—signal frequency for the samples after tensile loading.

## 3. Results

**Figure 4:**
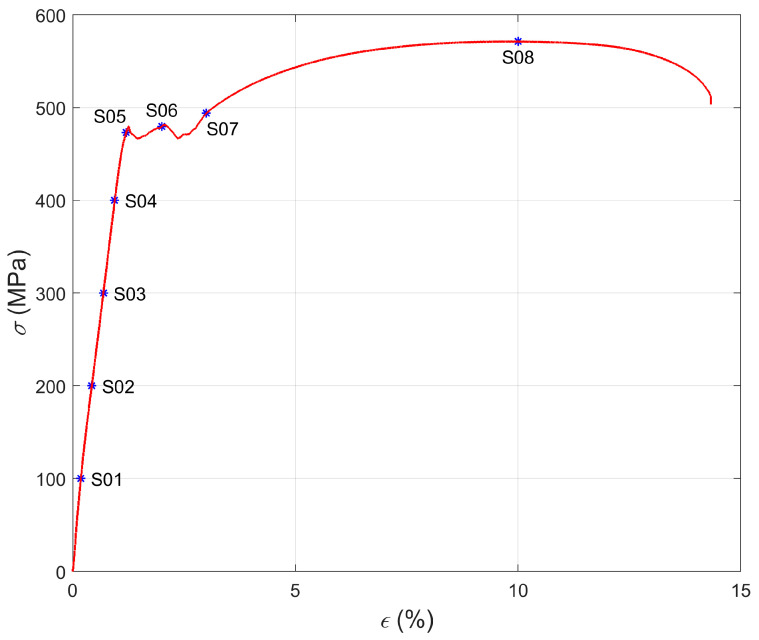
The stress–strain curve obtained for the sample made of S355. S01–S08—eight samples loaded to a different degree in elastic and plastic regions’ volume depicted on the curve.

This experiment was performed according to the following methodology. Eight samples (S01–S08) made of S355 were magnetized to record a quasi-sinusoidal pattern. Next, the 2D distribution of the magnetic field caused by the residual magnetization of the sample was measured using a magnetometer. Subsequently, each sample was loaded to a different degree in elastic and plastic regions’ volume using an Instron Universal Testing machine (Figure 4 and Table 1). In order to investigate possible changes in the magnetization pattern, the magnetic field was measured once again. The signals measured for each sample before and after stress-loading were stored and used to prepare plots presented in this section.

**Figure 5:**
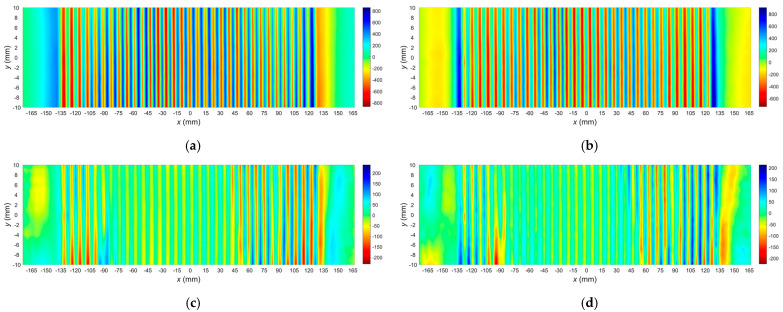
Results of 2D measurements of the magnetic field in the case of sample S05. (**a**) Component *B_x_* before tensile loading; (**b**) component *B_z_* before tensile loading; (**c**) component *B_x_* after tensile loading; (**d**) component *B_z_* after tensile loading.

The measurements of the magnetic field changes were carried out following the methodology described in Section 2. As a result, two sets of two-dimensional signals for each sample (S01–S08) were obtained: the first plot for the specimen before tensile loading and the second for the specimen after tensile loading. Figure 5 shows examples of two-dimensional signals measured in both cases for the sample S05. Similar graphs obtained for other samples were omitted because they would increase the article’s length without introducing important information. The plots show only two components *B_x_* and *B_z_* because the third component, *B_y_*, was a small amplitude signal unused for evaluation.

**Figure 6:**
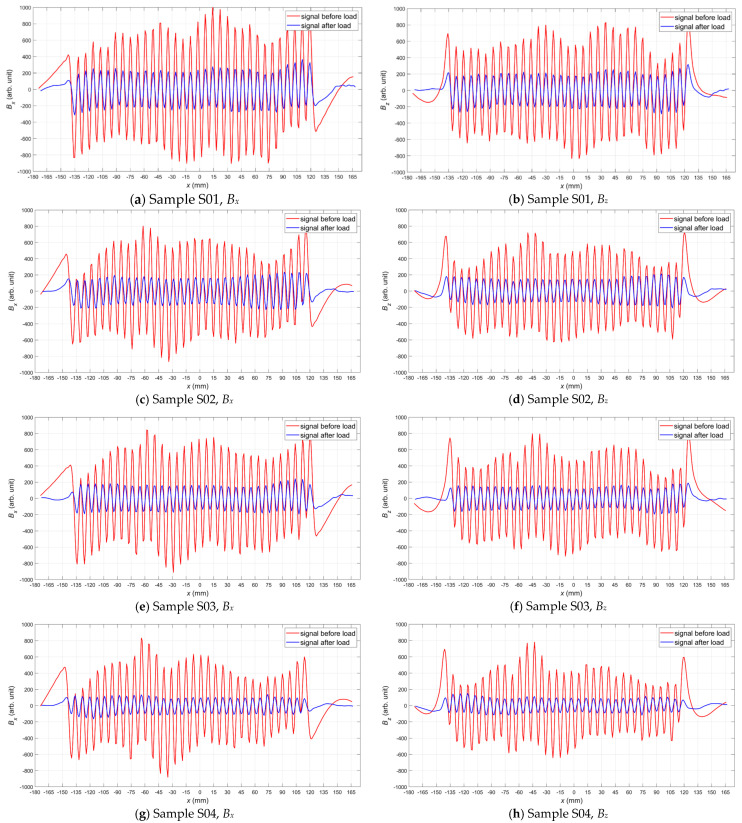

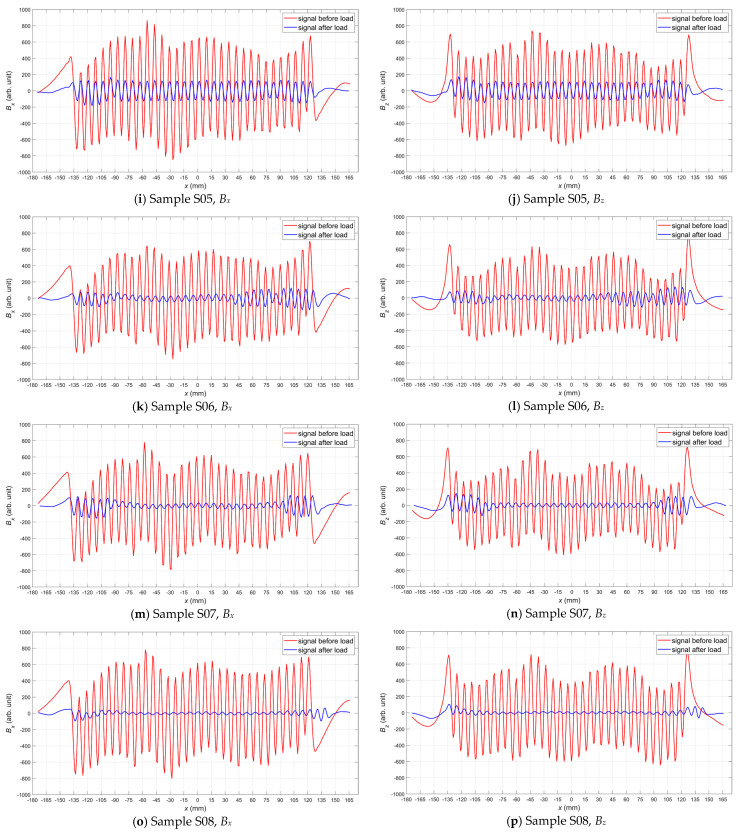
Components of the magnetic field measured for the magnetized samples before (red line) and after tensile loading (blue line): (**a**) *B_x_* for sample S01, (**b**) *B_z_* for sample S01, (**c**) *B_x_* for sample S02, (**d**) *B_z_* for sample S02, (**e**) *B_x_* for sample S03, (**f**) *B_z_* for sample S03, (**g**) *B_x_* for sample S04, (**h**) *B_z_* for sample S04, (**i**) *B_x_* for sample S05, (**j**) *B_z_* for sample S05, (**k**) *B_x_* for sample S06, (**l**) *B_z_* for sample S06, (**m**) *B_x_* for sample S07, (**n**) *B_z_* for sample S07, (**o**) *B_x_* for sample S08, (**p**) *B_z_* for sample S08.

In order to straightforwardly demonstrate the usability of the proposed method, the analysis was limited only to one-dimensional signals taken from the central part of the samples. The average signals of the *x* and *z* magnetic field components were calculated for each sample. Plots of the averaged signals for all samples before and after tensile loading are shown in Figure 6. The plots depict variations in the amplitude of the components depending on the sensor position along the *x*-axis. In the central part of the sample, an evident change in the signals can be observed.

Detailed analysis of the signals measured for samples S01–S05 (Figure 6) allows us to conclude that as the stress level increased, the magnetic field amplitude decreased in the central part of the measuring area, and frequencies *f_Bx_*, *f_Bz_* remained practically unchanged. In the case of the samples loaded over the yield point (S06–S08), the amplitudes and frequencies *f_Bx_*, *f_Bz_* of the signals measured after tensile loading significantly decreased compared to the parameters measured before tensile loading (Figure 6).

**Figure 7:**
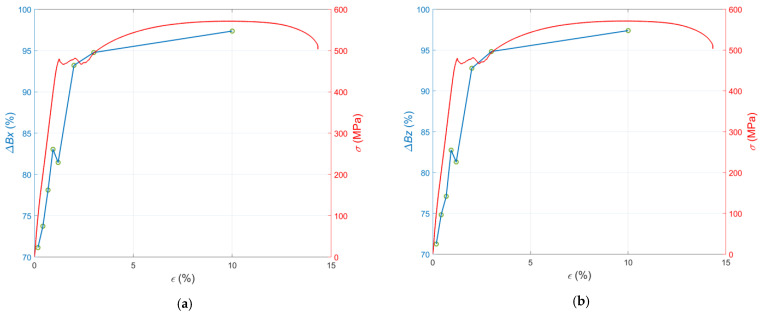
Relative mean changes in the magnetic field in the case of the samples S01–S08 plotted versus the strain: (**a**) component Δ*B_x_*; (**b**) component Δ*B_z_*.

Evaluating the condition of samples based solely on direct observation of the signal before and after tensile loading can be problematic due to the minor differences. For this reason, characteristic parameters were determined, and additional charts were prepared to visualize the changes taking place. First, the relative change in the magnetic field amplitude as a function of strain is presented (Figure 7). As can be seen from Figure 7a,b, the curve of the above relation consists of two parts separated by the point defining the elastic limit of the samples. For samples S01 to S04, the values increased approximately linearly. Then, starting with sample S04, the curve slopes sharply down to the value corresponding to the yield point sample S05. After the yield point was exceeded, the curve increases again to a point corresponding to the sample S08, but slower than its initial part. Thus, it can be concluded that an increase in the deformation level of the samples increased the value of the relative change in the residual magnetization.

**Figure 8:**
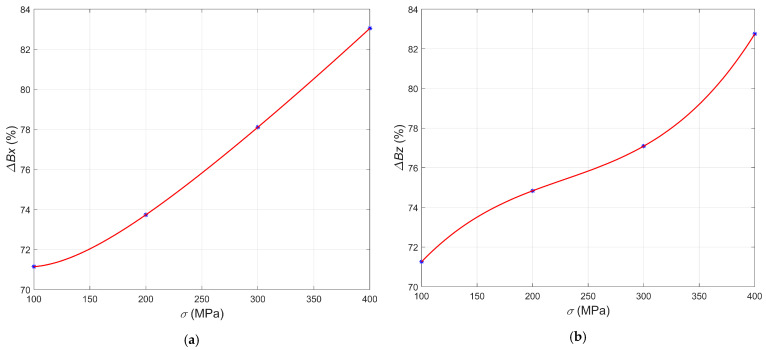
Relative mean changes in the magnetic field in the case of the samples S01–S04 plotted versus the stress: (**a**) component Δ*B_x_*; (**b**) component Δ*B_z_*.

**Figure 9:**
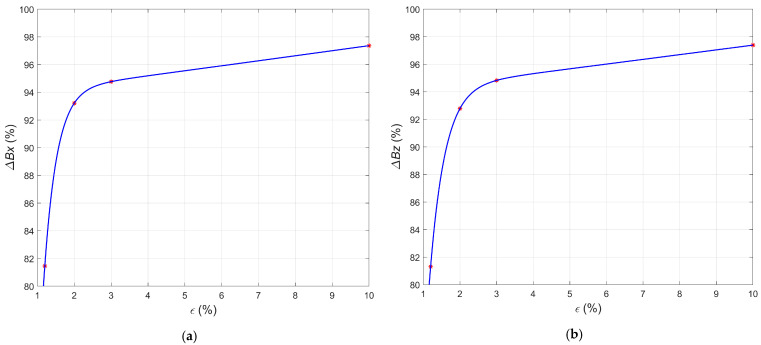
Relative mean changes in the magnetic field in the case of the samples S05–S08 plotted versus the strain. (**a**) component Δ*B_x_*; (**b**) component Δ*B_z_*.

Another two sets of plots contain the relative mean change in magnetic field *ΔB* as the function of applied stress *σ* for the samples S01–S04 (Figure 8) and strain *ε* for the samples S05–S08 (Figure 9), respectively. The reason for separating the parameter analysis of samples S01–S04 from samples S05–S08 is the change in mechanical properties at the point corresponding to sample S05. In the case of the first four specimens, the stresses induced an elastic deformation of the structure, and in the case of the remaining four specimens, plastic deformation was induced.

Figure 8a shows the relative mean change in the magnetic field Δ*B_x_*, Δ*B_z_* (Equation (3)) in the case of the samples S01–S04. Component Δ*B_x_* increased exponentially with the rise in the stress level. On the contrary, the curve for the component Δ*B_z_* (Figure 8b) rises slower and resembles the cubic polynomial. Due to the monotonicity of the curves, these graphs allow evaluating the sample conditions straightforwardly. Plots presented in Figure 9 show the relative mean change Δ*B_x_*, Δ*B_z_* in the magnetic field as the function of strain *ε* for the samples S05–S08.

**Figure 10:**
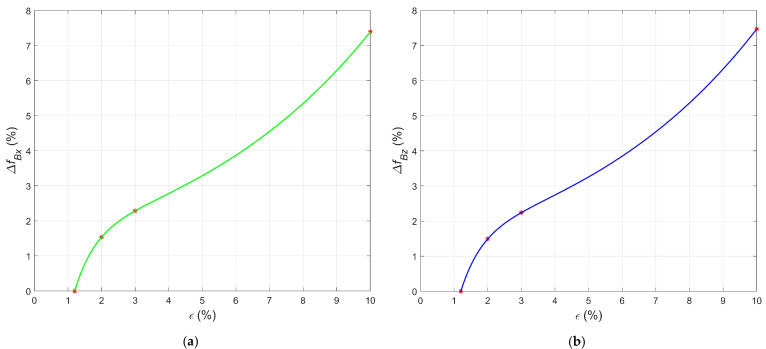
Relative changes in the signal frequency in the case of the samples S05–S08 plotted versus the strain. (**a**) Δ*f_Bx_*; (**b**) Δ*f_Bz_*.

Figure 9a,b indicates that the values of Δ*B_x_* and Δ*B_z_* increase exponentially with growing strain values. After passing the yield point corresponding to sample S05, the curve bends. This change is the transition from the elastic region through the yield point to the plastic region in the following samples. Plots showing the relative change in frequency Δ*f_Bx_,* Δ*f_Bz_* (Equation (4)) as a function of strain *ε* can also be used to evaluate the conditions of the samples S05–S08 (Figure 10). In the case of both components (*B_x_* and *B_z_*), the curves increase to the point of the maximum strain (sample S08). There is an inflection of the curve at the point corresponding to sample S07.

## 4. Discussion

The tests of the proposed method of nondestructive testing, which was presented in the previous section, covered several dozen samples made of the same material (S355) and should be treated as a first attempt to verify the suitability of the method.

The strictly defined signals (e.g., a sinusoid of a specific frequency) enable the use of dedicated filtering algorithms that effectively eliminate external disturbances. For example, a simple pass-band digital filter could eliminate a DC (Direct Current)) component from the signals presented in Figure 6. The parameters of the measured signals (e.g., the amplitude and frequency of the sine wave) can be determined by proven and reliable algorithms. These parameters allow for unambiguous identification of the material condition both in the elastic (Figure 8) and plastic regions (Figure 9). It should also be noted that external sources of DC magnetic fields have a limited impact on the results obtained in the proposed method. For example, such DC fields would not affect the frequency of the measured sinusoidal signal in any way. Such frequency change (Figure 10) is a very reliable parameter, but, unfortunately, it can only be observed in the case of samples loaded over the yield point.

The achieved results of the tests can generally be assumed as promising, and the method can help identify the condition of elements made of ferromagnetic materials subjected to loads. However, as the method is new, it is necessary to conduct further detailed tests to clarify existing doubts and improve the test procedure. The following aspects of the inspection procedure should be investigated and analyzed: the magnetization process, the residual magnetization measurement process, and the algorithms for analyzing the received signals.

One of the problems that has to be addressed is the decreasing magnetization of the tested elements over time. For this purpose, samples have been retained, and measurements will be repeated during the following year. Unfortunately, it was impossible to conduct comparative tests using other NDT methods before this time elapsed.

When comparing the results obtained by the proposed method with the results from other testing methods applied to very similar samples but made of SS400 steel, some significant differences can be observed. For example, in the case of the hysteresis loop observation method [36], unambiguous identification of the sample state is possible, but this method is less sensitive in the elastic range (measurements carried out after removing the load). Moreover, in this method, the spatial resolution is lower due to the larger dimensions of the transducer, and its implementation requires the use of a more complex measurement system.

Similarly, lower sensitivity in the elastic range can be observed in the case of the results obtained from the eddy current method [37] and the residual magnetization observation method with the GMR (Giant MagnetoResistance) transducer [38]. Additionally, measured parameters of the signals did not allow for unequivocal identification of the sample state as the same value was obtained for the samples before and after the yield point. A considerable advantage of the eddy current testing is the independence of the results on the magnetic history of the tested object.

The advantage of all the compared methods over the proposed Magnetic Recording Method is that there is no need to magnetize the sample with a specific pattern beforehand. Therefore, the proposed method can be applied only in some specific cases, for example, when it is necessary to constantly monitor crucial elements of the structure.

Due to the limited number of tests of a new method, it is not easy to make a reliable comparison with other methods. A comparison should also be made using the same or very similar samples. Unfortunately, during the experiment, it was not possible. Therefore, the comparison of the proposed method with the other nondestructive electromagnetic methods presented in Table 2 should be considered only as a preliminary attempt and will be updated after the next set of experiments.

## 5. Conclusions

The tightening of the requirements regarding the reliability of the structure creates the necessity for frequent inspections that will detect not only existing defects but also any changes that may indicate their occurrence. One such change is the residual stress distribution.

Several nondestructive testing methods can detect residual stress distribution and material changes due to stress. The authors assumed that it is also possible to analyze changes in the prerecorded magnetization of the tested element. Experiments have verified this, and the article proposes a Magnetic Recording Method that opens up new possibilities for monitoring critical structural elements.

Based on the results of the research conducted so far, it can be concluded that:

- The parameters (amplitude and frequency) of the quasi-sinusoidal pattern change significantly with the applied tensile stress, especially the amplitude in the elastic region and the frequency over the yield point.
- Regardless of the state, the load can be unequivocally determined based on formulated simple parameters.
- Additionally, the state after exceeding the yield point can be unequivocally determined based on changes in the amplitude of the signal and the frequency of the magnetization pattern.
- The obtained quasi-sinusoidal magnetization pattern is easy for later analysis.
- During the magnetization process, magnets can be placed at a relatively large distance (on the order of 1 mm) from the magnetized element.
- Further experiments are necessary to find the optimal and maximum distance between the magnets and the tested material.
- The proposed magnetization method can be used for flat surfaces.
- In the case of more complicated shapes of the tested element, it is necessary to make dedicated magnetizing systems.
- In order to obtain quasi-sinusoidal magnetization patterns on elements of larger sizes or to obtain a higher frequency of changes, it would be more effective to use a magnetizing head mounted on a motorized manipulator instead of magnets.
- The regularity of the magnetization pattern is not critical if the primary magnetization is measured and the signals are archived for normalization in later tests.
- While maintaining the signals measured after the magnetization process, the previous magnetization state of the sample is not important, but it is better to demagnetize the sample before magnetization to simplify the diagnostic process.

Despite the satisfactory initial test results, more research is needed to identify the method′s strengths and weaknesses and improve the testing process. It is planned to test other magnetization methods (e.g., using recording heads) to examine objects without a flat surface. Plans are underway to analyze the measured two-dimensional signals and utilize chosen statistics features to develop more criteria for the material condition assessments. An experiment will also be carried out to assess the effect of the passage of a long time period (over one year) on the sample′s magnetization state.

## Figures and Tables

**Table 1:** Tensile test results.

Sample	Stress (MPa)	Strain (%)
S01	100	0.18
S02	200	0.43
S03	300	0.69
S04	400	0.94
S05	473	1.20
S06	479	2.00
S07	494	3.00
S08	571	10.00

**Table 2:** Comparison of the Magnetic Recording Method with other magnetic methods.

	Metal Magnetic Memory	Hysteresis Loop	Barkhausen Noise	Magnetic Flux Leakage	Magnetic Particle	Residual Magnetization	Eddy Current	Magnetic Recording
Sensitivity to surface cracks	High	Low	Low	High	High	High	High	High
Sensitivity to subsurface cracks	High	Very Low	Very Low	Medium	Medium	Medium	High	High
Sensitivity to residual stress and plastic deformations(loading over the yield point)	High	High	High	Low	Very Low	High	High	High
Sensitivity to residual stress (loading below the yield point)	High	High	High	No	No	Medium	Low	High
Unambiguous identification of stress (loading below and over the yield point)	Medium	Medium	Low	No	No	Medium	Low	High
The necessity of preliminary preparation before operation	No	No	No	No	No	No	No	Magnetization of the pattern
The necessity of preliminary treatment before measurement	No	No	No	No	DC magnetization	DC magnetization	No	No
Influences of external DC fields during the measurement	Very High	Low	Low	Low	Medium	High	Very Low	Low/Medium
Influences of external AC fields during the measurements	Low	Low	High	Low	No	No	High	No
Influences of DC magnetization before the measurements	Very High	Low	Low	Low	Medium	Low	No	Low/Medium
Measurement speed	High	Low	Low	High	Medium	High	High	High
The complexity of the instrumentation	Low/Medium	High	High	Low	Very Low	Low	Medium	Low
Repeatability of the results	Low	Medium	Medium	High	High	High	Very High	High
Spatial resolution	High	Low/Very Low	Low/Very Low	High	Medium	High	Medium/High	High

## Data Availability

The data presented in this study are available on request from the corresponding author. The data are not publicly available due to a complicated structure that requires additional explanation.
